# Family Systems Considerations in Autism Spectrum Disorder After Prenatal Trauma and Prior Pregnancy Loss

**DOI:** 10.7759/cureus.114998

**Published:** 2026-08-22

**Authors:** Jonathan Bazile, Nadia Bernard, Teffera Y Ephraim, Ranwinder Kaur, Abhiroop Kaur Khera

**Affiliations:** 1 Obstetrics and Gynecology, State University of Haiti, Port-au-Prince, HTI; 2 Family Medicine, Care Promotion PC, Canton, USA; 3 Cardiac Surgery, St. Michael's Hospital, Toronto, CAN; 4 Internal Medicine, Sri Guru Ram Das University of Health Sciences, Amritsar, IND; 5 Internal Medicine, Dayanand Medical College and Hospital, Ludhiana, IND

**Keywords:** autism spectrum disorder, caregiver adaptation, family systems, neurodiversity, perinatal loss, prenatal trauma

## Abstract

We present the case of a four-year-old child evaluated for core features of autism spectrum disorder, whose family context included a history of second-trimester perinatal loss 14 months prior to conception and severe maternal motor vehicle trauma at 18 weeks’ gestation. This background coincided with parental hypervigilance, anticipatory grief, and complex caregiving dynamics, while early infancy was characterized by severe regulatory disturbances and sensory sensitivities that interacted with parental stress responses. Management utilized a dual approach combining neurodiversity-affirming developmental interventions for the child with Child-Parent Psychotherapy for the parents, where addressing the intersection of historical loss and developmental neurodivergence supported family co-regulation and functional communication gains for the child. This case illustrates how unresolved parental trauma may influence family adaptation following an autism diagnosis, demonstrating that incorporating trauma-informed, family-centered assessment alongside evidence-based developmental interventions may improve caregiver functioning and support the implementation of therapeutic strategies.

## Introduction

Autism spectrum disorder (ASD) is a neurodevelopmental condition rooted in intrinsic neurobiological and genetic mechanisms [1]. However, the day-to-day functional adaptation, developmental surveillance, and emotional climate of a neurodivergent child unfold within a specific family matrix [2]. When a child’s developmental trajectory is preceded or accompanied by severe parental stressors - such as acute prenatal physical trauma and unintegrated perinatal loss - the family system can encounter challenges related to chronic hypervigilance, anticipatory grief, and altered parental reflective functioning [3].

Extensive developmental literature highlights that family systems frequently navigate profound psychological readjustments following a neurodevelopmental diagnosis, wherein parental coping mechanisms directly moderate household stress dynamics [3-4]. Rather than altering the fundamental neurodevelopmental trajectory of autism, parental trauma history heavily shapes how families perceive, process, and respond to early behavioral differences [5]. Herein, we present a case report illustrating the intersection of prenatal trauma exposure, prior perinatal loss, and family systems adaptation in early childhood ASD, demonstrating the utility of an integrated, family-centered intervention model [3-6].

## Case presentation

A four-year-old male was referred to a developmental clinic because of delayed expressive language, restricted and repetitive behaviors, and social-communication differences consistent with ASD [7].

The family history was notable for two significant stressors. Approximately 14 months prior to conceiving the child, the mother experienced a second-trimester miscarriage, which contributed to heightened anxiety during the subsequent pregnancy. In addition, at 18 weeks' gestation, she was involved in a high-impact motor vehicle collision requiring prolonged hospital observation following localized abdominal trauma. Following the accident, she was evaluated by a treating mental health professional and diagnosed with acute stress disorder according to the Diagnostic and Statistical Manual of Mental Disorders, Fifth Edition (DSM-5) criteria, based on the presence of intrusive trauma-related memories, heightened anxiety, hyperarousal, and trauma-related somatic symptoms occurring during the acute post-trauma period [7].

During infancy, the child demonstrated early regulatory difficulties, including severe colic, disrupted sleep patterns, and sensory sensitivities. By age 2.5 years, expressive language remained limited, with frequent echolalic speech patterns [7]. Additional concerns included repetitive object alignment, hand-flapping during periods of sensory overload, and pronounced distress during routine changes [3].

Family functioning was evaluated through a structured family systems clinical assessment consisting of parent interviews focused on family organization, coping patterns, caregiver stress, and parent-child interactions. This assessment identified a complex pattern of adaptation within the household [4].

Maternal trauma history appeared to affect her interpretation of some of the child's self-regulatory behaviors, such as rhythmic rocking and vocalization patterns, which she associated with memories of her prior trauma. Concurrently, the father responded to family stress through increasingly rigid household routines and protective behaviors, which may have contributed to elevated family stress levels [3,4].

Management consisted of a coordinated multimodal intervention plan. The parents participated in weekly Child-Parent Psychotherapy sessions designed to address the psychological impact of prior reproductive loss and traumatic stress exposure [6]. Simultaneously, the child received individualized play-based developmental services emphasizing sensory accommodations, predictable visual supports, and functional communication development. Family counseling sessions focused on supporting parental understanding of the child's neurodevelopmental profile and promoting adaptive coping strategies.

At a 12-month follow-up, reassessment using the Vineland Adaptive Behavior Scales demonstrated improvement in functional expressive communication and fewer reported behavioral meltdowns [8]. Parents also showed reductions in self-reported anxiety and trauma-related symptoms as measured by the Generalized Anxiety Disorder-7 (GAD-7) and PTSD Checklist for DSM-5 (PCL-5). Because multiple interventions were delivered concurrently, the relative contribution of any individual intervention to the observed changes cannot be determined.

The child's ASD is most appropriately understood as a neurodevelopmental condition associated with underlying biological and genetic mechanisms [1,7]. The maternal trauma history and prior pregnancy loss should not be interpreted as causal factors in the child's autism diagnosis. Rather, these experiences may have influenced caregiver perceptions, stress responses, and family adaptation following recognition of developmental concerns [2-6].

## Discussion

This case highlights the importance of distinguishing between the intrinsic neurobiology of autism and the family's adaptation to an autism diagnosis [1-4]. Although ASD is an independent neurodevelopmental condition, parental interpretation of developmental differences may be influenced by previous experiences of trauma, grief, or loss [5,6].

In this family, prior reproductive loss and prenatal trauma coincided with significant caregiver anxiety and hypervigilance. The child's sensory and regulatory challenges occurred within this context, contributing to complex family stress dynamics. While improvements were observed following implementation of developmental interventions, family counseling, and Child-Parent Psychotherapy, the simultaneous delivery of these services prevents determination of their individual effects.

These observations are consistent with literature demonstrating associations between caregiver psychological well-being, family functioning, and adaptation to childhood developmental conditions [2,3]. The findings further suggest that integrating assessment of family stressors into developmental evaluations may provide a more comprehensive understanding of factors influencing treatment implementation and caregiver functioning.

Because this report describes a single patient and family, the findings should be interpreted as exploratory and hypothesis-generating rather than evidence of causation or treatment effectiveness.

Clinical implications 

Integrated Assessment

When clinically indicated, developmental evaluations may benefit from consideration of caregiver trauma history, reproductive loss, or significant psychosocial stressors, particularly when concerns regarding family functioning or caregiver distress are present [5].

Family-Centered Intervention

For some families, addressing caregiver psychological needs alongside developmental interventions may be beneficial and should be considered when clinically appropriate [6].

## Conclusions

This case highlights the complex presentation of ASD within the broader context of family stress, prior reproductive loss, and prenatal trauma exposure. While these factors should not be interpreted as causes of autism, they may influence caregiver perceptions, stress responses, and family adaptation following developmental diagnosis.

The case underscores the value of a trauma-informed, family-centered assessment framework that considers both the needs of the child and the caregiving environment. Improvements in child functioning and caregiver well-being were observed during a period in which multiple interventions were provided concurrently; however, the single-case design does not permit conclusions regarding causality or the effectiveness of any specific intervention. Future research using larger samples and controlled study designs is needed to better understand how family-centered and trauma-informed approaches may support families of autistic children and facilitate implementation of evidence-based developmental interventions.
